# Editorial: Intrinsic Clocks

**DOI:** 10.3389/fneur.2018.00068

**Published:** 2018-02-16

**Authors:** Timo Partonen, Daniela D. Pollak

**Affiliations:** ^1^Department of Public Health Solutions, National Institute for Health and Welfare (THL), Helsinki, Finland; ^2^Department of Neurophysiology and Neuropharmacology, Medical University of Vienna, Vienna, Austria

**Keywords:** circadian rhythm, depression, marine biology, mouse model, plasticity, seasonality, small-molecule, systems biology

**Editorial on the Research Topic**

**Intrinsic Clocks**

The existence of living organisms on our planet has been dependent on and co-evolved with the foreseeable variations in environmental conditions oscillating over recurring periods. All species have responded to these exogenous rhythms by developing endogenous clocks that allow for an approximate, but reliable estimation of the periodic changes and elicit corresponding adaptive processes.

The importance of these mechanisms for health and disease has been highlighted by the 2017 award of the Nobel Prize in Physiology or Medicine to Jeffrey C. Hall, Michael Rosbash, and Michael W. Young for their discoveries of the genetic control of the daily biological rhythm. They explained in molecular terms how the gene named as *period* contributed to the emergence (eclosion from the pupal case) rhythm of a population and to the locomotor activity of individual flies (*Drosophila melanogaster*). The key to the explanation was the discovery of transcription-translation feedback loops of the so-called “clock genes.”

This research topic on *Intrinsic Clocks* which appeared earlier comprises a well-balanced collection of original research and review articles on endogenous rhythms from seasonal and monthly to daily and hourly oscillations in different experimental model systems with analytical approaches from systemic to cellular and molecular levels.

Serchov and Heumann in their review focus on the role of Ras, an enzyme which hydrolyzes guanosine triphosphate and dependent intracellular signaling cascades in the regulation of the circadian rhythm in mice. They elegantly summarize how Ras activity forms a molecular bridge between entrainment of the suprachiasmatic nucleus that is the master clock in the brain and synaptic plasticity in dependent brain regions, such as the hippocampus, and corresponding functions. The extensive study by Chiang et al. specifically investigated rhythmic alterations in the murine hippocampus. They characterized the protein phosphorylation using a mass spectrometry approach with which they provided large-scale quantitative analysis of the daily oscillation of hippocampal phosphorylation events over a range of biological pathways. The hippocampus is a key focus also in the review by Urs Albrecht. It features the role of circadian proteins in the control of adult hippocampal neurogenesis, reciprocally implicated in depression and antidepressant responses. He discusses neurobiological mechanisms implicated in the pathogenesis of mood disorders, such as monoaminergic neurotransmission and stress response by the hypothalamic–pituitary–adrenal axis. The hypothalamus and the pituitary are further involved in seasonal cycles as highlighted in the review by Lewis and Ebling who elaborate in detail on the role of tanycytes, pituitary radial glial cells, in the regulation of circannual clocks in hamsters. They provide evidence supporting their hypothesis that tanycytes serve as central organizers of seasonal rhythms in the adult hypothalamus. Raible et al. present in their review on marine animals the current insight in the cellular mechanisms in molecular detail the monthly or semi-monthly rhythms. They express their worry about light pollution and further review the relevance of circalunar rhythms to mammalian physiology and reproduction in specific. They speculate that these rhythms may be the remnant of evolutionary ancient clocks, which were uncoupled from a natural entrainment mechanism.

Bourguignon and Storch summarize recent findings of the cellular substrate and mechanism, which generate locomotor activity with periods of 2–6 h. Such rhythms are normally integrated with circadian rhythms, but often lack the period stability and expression robustness. They further review the concept of the dopaminergic ultradian oscillator and show that ultradian locomotor rhythms rely on cells in the brain using dopamine for transmission. Intriguingly, Monje et al. report in their study on interleukin-6 knockout mice that the ultradian locomotor rhythm was impaired under both light-entrained and free-running conditions, whereas the circadian period and the level of locomotor activity as well as the phase shift response to light exposure at night remained normal. During the day, *Cry1* and *Bhlhe41* expression levels were increased whereas those of *Nr1d2* were decreased in the hippocampus. Liu and Zhang first created mutants of cryptochrome circadian clock 1 (Cry1) protein at potential phosphorylation sites and conducted thereafter a screen in *Cry1*/*Cry2* double deficient cells. They targeted at identifying mutations that disrupted circadian rhythms. They found that these single amino acid substitutions changed not only the circadian period, but also repression activity, protein stability, or cellular localization of the protein. Concerning the circadian period, Narasimamurthy and Virshup elucidate in their review the molecular mechanisms that regulate an enigma of the clock. Unlike other chemical reactions, the output of the clock as measured with the period remains nearly constant with fluctuations in ambient temperature. This is called as temperature compensation. The key lies especially in the mechanism that controls the stability of period circadian clock 2 protein. Clock-enhancing small molecules have become of particular interest as candidate chronotherapeutics, since there is a close association of circadian amplitude dampening with progression of chronic diseases, especially that of mood disorders. Gloston et al. present in their review an update of the regulatory mechanisms of circadian amplitude and the current status of these small molecules of therapeutic interest. Millius and Ueda introduce the readers to study of biology which takes advantage of engineering and mathematical tools to model and test the behaviors of the intrinsic clocks. It has evolved through the development of both wet lab and *in silico* work. The goal here is to understand the clocks that are made up of a range of complex properties of cells, tissues, and organisms.

The cross-section of studies comprised in this research topic on *Intrinsic Clocks* highlights the vibrant scientific activity in the field of the investigation of endogenous biological rhythms and their relevance for physiology and pathology.

## Author Contributions

TP and DP planned and wrote the manuscript together.

## Conflict of Interest Statement

The authors declare that the research was conducted in the absence of any commercial or financial relationships that could be construed as a potential conflict of interest.

